# Mycobacterium tuberculosis and Mycobacterium avium Coinfection in an Immunocompetent Patient

**DOI:** 10.7759/cureus.63108

**Published:** 2024-06-25

**Authors:** Sailesh Karki, Sagar Pandey, Nabin K C, Arjun Mainali, Muhammad N Pasha, Harish J Patel

**Affiliations:** 1 Internal Medicine, One Brooklyn Health/Interfaith Medical Center, Brooklyn, USA; 2 Pulmonary and Critical Care Medicine, One Brooklyn Health, Brooklyn, USA

**Keywords:** mycobacterium tuberculosis, mycobacterium avium complex, nontuberculous mycobacteria (ntm), azithromycin allergy, coinfection, tuberculosis

## Abstract

Despite the increasing incidence of simultaneous mycobacterial and non-mycobacterial tuberculosis (TB) infection, little literature is available exploring the topic. Here, we present a case of a 22-year-old female diagnosed with pulmonary TB for four months with simultaneous multiple sputum cultures positive for non-tuberculous mycobacteria (NTM). Computed tomography of the chest without contrast reported linear areas of scarring involving both lung apices, more prominent on the left side. The patient completed intensive phase treatment for TB and is currently on isoniazid and rifampin with a referral to an infectious disease specialist for recommendations on treatment of *Mycobacterium avium* regimen in view of azithromycin allergy (intense cough and rash). While the coexistence of NTM is commonly attributed to colonization, differentiating colonization from disease is crucial considering the long duration of treatment, potential drug toxicity, risk of drug resistance, and significant cost of treatment. Clinical, microbiological, and radiological evidence should be considered for diagnosis of TB and NTM coinfection and expert consultation should be sought in formulating the treatment plan.

## Introduction

*Mycobacterium avium* complex (MAC) is comprised of multiple non-tuberculous mycobacteria (NTM) that cause pulmonary disease, skin and soft tissue infections, musculoskeletal infections, disseminated disease, catheter-associated disease, and lymphadenitis [1]. Two major clinical manifestations of *Mycobacterium avium* pulmonary disease have been identified. The fibrocavitary subtype is rapidly progressive and usually develops in middle-aged male smokers [2]. In contrast, the nodular bronchiectatic subtype classically develops in non-smoker females and is also known as Lady Windermere syndrome [1,3]. Disseminated, multi-organ involvement occurs in immunocompromised patients, including patients taking immunosuppressive medications and people with acquired immunodeficiency syndrome (AIDS) [4]. The incidence of NTM has been increasing in developed countries, with a simultaneous decrease in *Mycobacterium tuberculosis* (MTB) infection. This has been partially attributed to improved diagnostic methods for NTM [5]. The coinfection of MTB and *Mycobacterium avium* in an immunocompetent patient in a developed country is rare and presents a diagnostic and clinical challenge. This case report discusses a unique presentation of such a coinfection in a young immunocompetent female.

## Case presentation

**Figure 1:**
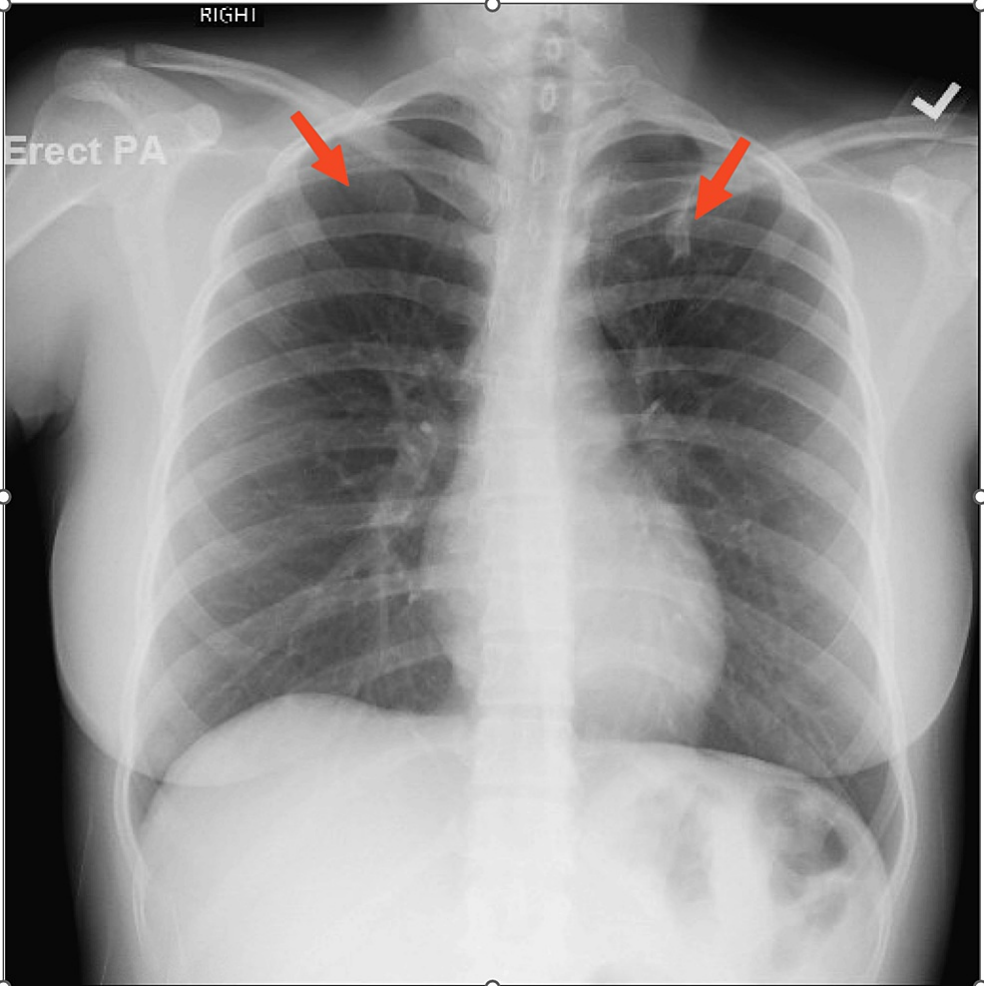
Chest X-ray demonstrating apical lobe scarring/atelectasis (red arrows).

**Figure 2:**
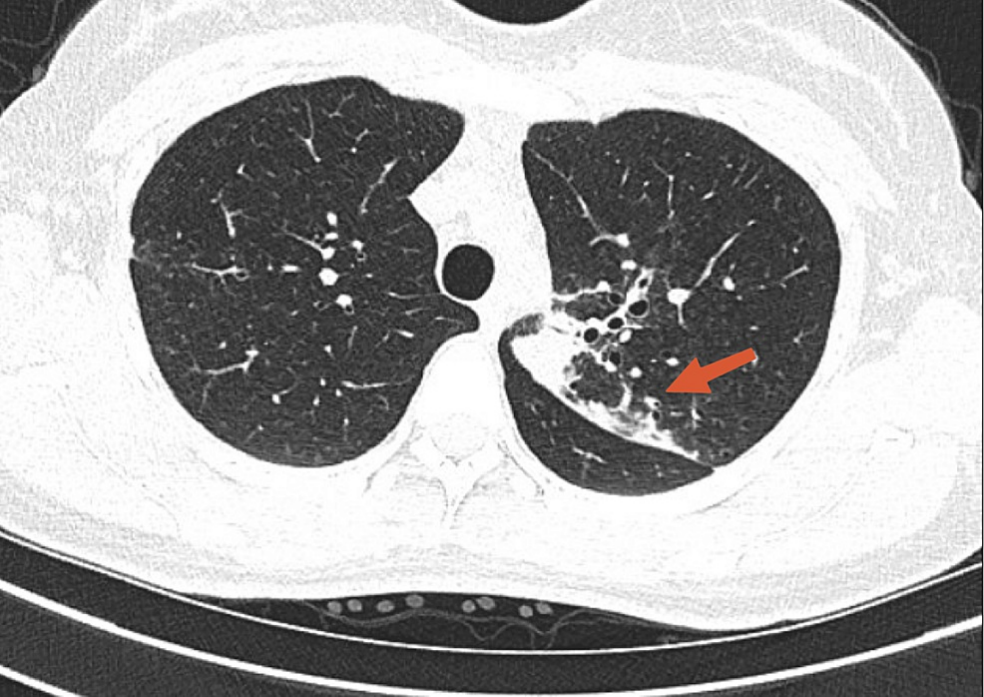
CT of the chest without contrast demonstrating linear areas of scarring involving both lung apices and more prominent on the left side (red arrow).

A 22-year-old female diagnosed with pulmonary tuberculosis for four months was referred to the pulmonary clinic by the patient’s primary care physician due to multiple sputum cultures positive for MAC. The patient reported that the symptoms started as night sweats and sore throat. The symptoms had been started two months before the initial diagnosis of pulmonary tuberculosis. However, she denied any history of fever, cough, hemoptysis, weight loss, chest tightness, or positive contact history. She denied smoking, consuming alcohol, or using any illicit drug. Past medical history was significant for a diagnosis of latent tuberculosis at 15 years of age, which was treated with eight months of isoniazid and vitamin B6. The patient had no significant travel history. On examination, the patient’s vital signs were stable. Bilateral bronchial breath sounds were heard on chest auscultation. The rest of the examination findings were within normal limits. Chest X-ray (CXR) showed bilateral apical lobe scarring/atelectasis (Figure 1). Computed tomography of the chest without contrast corroborated the CXR findings and reported linear areas of scarring involving both lung apices, more prominent on the left side (Figure 2).

The patient was diagnosed with pulmonary tuberculosis when she was admitted to the hospital for upper respiratory tract symptoms, which were not resolved on antibiotics. Workup was done for pulmonary tuberculosis, including sputum cultures and bronchoscopy sputum specimens, which tested positive for tuberculosis. The patient was, however, tested negative for HIV. She was started on intensive phase treatment for pulmonary tuberculosis, i.e., isoniazid, rifampin, pyrazinamide, and ethambutol, along with pyridoxine. Ethambutol and pyrazinamide had to be dose adjusted because of adverse effects to the medications. The patient was in the continuation phase of treatment with isoniazid and rifampin when she presented to the pulmonary clinic. On presentation to our clinic, she had decreased night sweats but persistent sore throat. Multiple sets of sputum and bronchial fluid specimens from bronchoscopy tested positive for mycobacterial tuberculosis and nontubercular mycobacteria (MAC). Lastly, the patient reported an allergy to azithromycin in the form of intense cough and rashes. Therefore, the patient was referred to an infectious disease specialist for expert opinion on the treatment regimen for the patient for coinfection of MTB with MAC in the setting of macrolide allergy.

## Discussion

MAC organisms are ubiquitous in many environmental sites and are present in water, soil, and animals. The organism is thought to be acquired by inhalation or ingestion. There is no evidence for human-to-human transmission [6]. Differentiating colonization from disease is crucial in MAC considering the long duration of anti-microbial treatment, potential drug toxicity, and significant cost of treatment [7]. Detection of MAC in more than one sputum sample is recommended to rule out the possibility of contamination. Moreover, clinical and radiographic presentation has to be considered by the clinician, in addition to microbiological identification of MAC to avoid misinterpreting colonization as a true infection [1,7]. Our patient had an atypical presentation with a sore throat and night sweats. Additionally, radiography demonstrating apical scarring and tree bud opacities and multiple positive MAC results were supportive of MAC clinical disease.

Fujita et al. demonstrated concurrent infections with MAC involving microorganisms like methicillin-sensitive *Staphylococcus aureus* (MSSA), *Pseudomonas aeruginosa*, and *Aspergillus*. There was an increased prevalence of concurrent infection in those with chronic obstructive pulmonary disease (COPD) [8]. In a subsequent study, the coinfections were shown not to have any impact on the therapeutic efficacy of MAC [9]. Another study looked into the impact of concomitant infection by fungal species like *Aspergillus fumigatus*, *Histoplasma capsulatum*, and *Cryptococcus neoformans*. Patients with fungal co-infections were found to have a poorer prognosis [10]. Further studies are required to determine the impact of MTB coinfection on the prognosis of patients with MAC.

Coinfection with *Mycobacterium avium* may have important implications in selecting anti-microbial therapy. Bazzi et al. described a case where coinfection with MAC resulted in an incorrect interpretation of MTB as rifampin-resistant. This occurred because the test (GeneXpert) simultaneously identified the rifampicin resistance gene of *M. avium* and detected the tuberculosis genes of MTB [11]. This points to the need for a multiplex polymerase chain reaction (PCR)-based mycobacterial detection system in areas with a high prevalence of MAC [12]. Treatment of MAC also has implications for lung function. In a study evaluating the coinfection of MAC with MTB, anti-MAC therapy was found to improve pulmonary function tests in patients who had abnormal pulmonary function at baseline [13].

## Conclusions

*Mycobacterium tuberculosis* and *Mycobacterium avium* coinfection is increasing in incidence. Differentiating colonization from the disease of NTM is critical for the optimal treatment of patients, decreasing side effects, and reducing the risk of drug resistance.
